# StopApp: Using the Behaviour Change Wheel to Develop an App to Increase Uptake and Attendance at NHS Stop Smoking Services

**DOI:** 10.3390/healthcare4020031

**Published:** 2016-06-08

**Authors:** Emily Anne Fulton, Katherine E. Brown, Kayleigh L. Kwah, Sue Wild

**Affiliations:** 1 Center for Technology Enabled Health Research (CTEHR), Coventry University, Coventry CV1 5FB, UK; Emmie.fulton@coventry.ac.uk (E.A.F.); Kayleigh.kwah@coventry.ac.uk (K.L.K.); 2 Public Health Warwickshire, Warwickshire County Council, Warwick CV34 4RL, UK; suewild@warwickshire.gov.uk

**Keywords:** behaviour change, COM-B, behaviour change wheel, stop smoking services, web-app, eReferral

## Abstract

Smokers who attend NHS Stop Smoking Services (SSS) are four times more likely to stop smoking; however, uptake has been in decline. We report the development of an intervention designed to increase uptake of SSS, from a more motivated self-selected sample of smokers. In Phase 1 we collected data to explore the barriers and facilitators to people using SSS. In Phase 2, data from extant literature and Phase 1 were subject to behavioural analysis, as outlined by the Behaviour Change Wheel (BCW) framework. Relevant Behaviour Change Techniques (BCTs) were identified in order to address these, informing the content of the StopApp intervention. In Phase 3 we assessed the acceptability of the StopApp. Smokers and ex-smokers identified a number of barriers to attending SSS, including a lack of knowledge about what happens at SSS (Capability); the belief that SSS is not easy to access (Opportunity); that there would be ’scare tactics’ or ‘nagging’; and not knowing anyone who had been and successfully quit (Motivation). The ‘StopApp’ is in development and will link in with the commissioned SSS booking system. Examples of the content and functionality of the app are outlined. The next phase will involve a full trial to test effectiveness.

## 1. Introduction

Smoking remains a leading cause of mortality and morbidity worldwide [1]. Smoking is prevalent across age groups, cultures, and communities, although it is more common in more socially deprived groups [2]. Reducing the prevalence of smoking in the United Kingdom is an ongoing challenge for public health. National Health Service (NHS) Stop Smoking Services (SSS) were established in England and Wales in 2000 (see www.nhs.uk/smokefree), and offer the most cost effective interventions for supporting smokers to stop [3]. Smokers are four times more likely to stop smoking with their help than when attempting alone [4]. However, attendance in recent years has been in decline [5], despite the fact that approximately two thirds of smokers report that they want to quit [1]. Between April 2014 and March 2015, 23% fewer smokers set a quit date than in the previous year, and this is the third consecutive year that numbers have fallen [5]. This may be attributed, at least in part, to the increased popularity of electronic cigarettes, with evidence that they are less harmful than smoking [6,7]. Some concern remains about the long-term health implications of their use [8], but SSS are advised by Public Health England to support smokers who want to use e-cigarettes to help them to stop, and it is possible that the combination of attending SSS and using e-cigarettes is more effective than using e-cigarettes alone.

Despite debate and opposition, as pressures to make cost savings in the NHS continue, there is uncertainty about whether SSS will continue to be commissioned on the same scale in the future. Where fewer services exist, however, it will be increasingly important to maximize the efficiency of such services by focusing resources on those who are most motivated, know what to expect, and intend to quit. Indeed, evidence suggests that smokers seeking help independently tend to be more successful at stopping in the long term compared to those who are proactively recruited by others, such as health professionals [9].

People from more deprived backgrounds are more likely to smoke. Despite reporting a similar desire to stop smoking, however, rates of smoking in these groups have undergone little or no change in the past decade despite overall rates for all smokers decreasing [10]. Although the literature suggests that SSS are serving more deprived groups, their representation varies across services [11]. The suspension of the tobacco control mass media campaign in April 2012 did not affect attendance at SSS [12], suggesting that this is not an effective intervention for addressing the decline in uptake. However, a recent study [13] found that giving booklets about the effectiveness of SSS increased attendance at SSS by 30%. Furthermore, the use of proactive recruitment to SSS via letters from a patient’s general practitioner (GP) has shown promising results [14], with a current evaluation assessing the addition of personalized risk information and additional taster sessions [15]. Interventions are needed that address the barriers to accessing and attending SSS, particularly for those from lower socioeconomic backgrounds. Therefore, consideration should also be given to proactively targeting more deprived smokers, with methods used to encourage uptake and the content of smoking cessation interventions, recognizing that SSS support will enhance attempts to quit with the use of e-cigarettes if this is their preferred method.

## 2. A Theoretically Driven Behaviour Change Intervention

**Figure 1:**
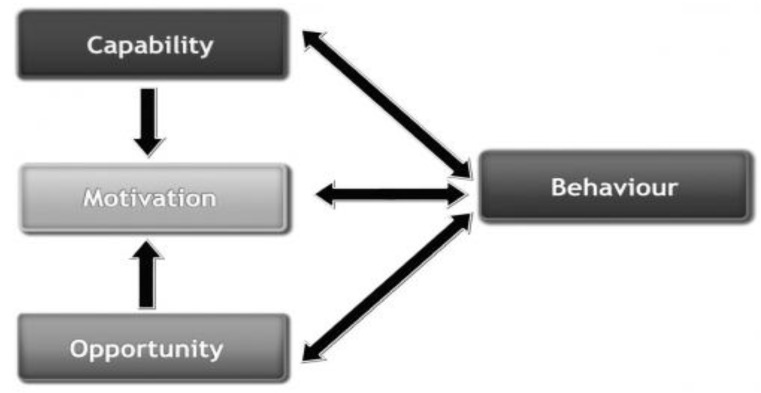
The COM-B Model (Michie, van Stralen & West, 2011) [20].

A systematic review identified online interventions based on more in-depth use of theory, and consisting of a greater number of ‘Behaviour Change Techniques’ (BCTs), as most effective at changing health behaviour [16]. Theoretically driven interventions developed to support smoking cessation have often been theory specific and typically based on the transtheoretical model (e.g., [17,18]). Because no one theory of behaviour change can be said to offer a complete explanation of behaviour, however, it is arguably more appropriate to select approaches that are theory agnostic [19] and encompass a range of systematically mapped theoretical concepts. The Behaviour Change Wheel (BCW) [20] is a theoretically driven framework based on multiple models of health behaviour, designed to enable the systematic development of interventions for supporting behaviour change. It is underpinned by the ‘COM-B model’ (a theoretical model based on 19 existing frameworks of behaviour change identified in a systematic review to understand the predictors of behaviour change). The model consists of three necessary conditions for a given ‘Behaviour’ to occur: (1) ‘Capability’ (psychological/physical); (2) ‘Opportunity’ (physical/social); and (3) ‘Motivation’ (reflective/automatic) (see Figure 1). Where more detail is needed, Capability, Opportunity, and Motivation can be sub-divided into 14 further constructs (domains) within the Theoretical Domains Framework (TDF). Exploration of a given behaviour in relation to the COM-B and/or TDF components helps identify which psychological determinants need to be addressed in order to achieve behaviour change. The BCW framework then supports the selection of intervention functions and policy categories, if needed, and specific BCTs from the Behaviour Change Technique Taxonomy (BCTTv1) [21] on which to base intervention content.

## 3. Aim

To explore the barriers to and facilitators of smokers accessing SSS in order to identify: (i) the target behaviour(s) for an intervention to increase uptake at SSS, and (ii) the design of an intervention to achieve change in the target behaviour(s).

## 4. Experimental Section and Results

**Figure 2:**
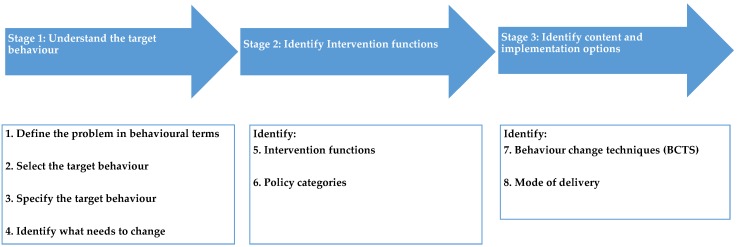
The BCW Intervention design process (Michie, Atkins & West, 2014 [22]).

The process of intervention development using the BCW is outlined in detail [22] and has been applied extensively elsewhere (e.g., [23]). It involves three key stages: Stage 1: Understanding the behaviour and identifying what needs to change; Stage 2: Identifying Intervention Functions; and Stage 3: Identifying content and the relevant ‘Mode of Delivery’ for the intervention (see Figure 2). These can be further subdivided into key steps including (i) defining the problem in behavioural terms; (ii) selecting the target behaviour; (iii) specifying the target behaviour; (iv) identifying what needs to change; (v) identifying appropriate intervention functions; (vi) identifying policy categories; (vii) identifying behaviour change techniques; and (viii) determining the mode of delivery.

### 4.1. Stage 1—Understand the Behaviour and Identify What Needs to Change

#### 4.1.1. Step 1—Define the Problem in Behavioural Terms

Step 1 involves defining the problem in behavioural terms, being specific about the individual, group, or population involved in the behaviour, and the behaviour itself. Although knowledge about the healthcare implications of smoking is widespread, approximately 19% of the UK population smokes. Supporting people to stop smoking long-term remains a challenge, even for those motivated to quit [24]. There is currently a lack of interventions aimed at increasing the uptake of attendance at SSS. As attending SSS significantly increases the chances of success, increasing the uptake of SSS was chosen as the focus of this intervention.

#### 4.1.2. Step 2—Selecting the Target Behaviour

Based on the decision to focus on increasing the uptake of SSS to address the overall behaviour that needs to change (stopping smoking), step 2 involves considering all the possible behaviours that could be targeted in the interventions to increase uptake at SSS. In order to explore possible target behaviours, we conducted discussions with an expert panel from SSS, Tobacco Control and Public Health; and a lay panel of smokers and ex-smokers, some of whom had previously used SSS.

Examples of possible target behaviours included:

■ Smokers to book an initial stop smoking advisor appointment.
■ Smokers to attend a stop smoking advisor appointment.
■ Healthcare professionals to (i) signpost and (ii) refer smokers to SSS.
■ Other professionals who come into contact with people who smoke, e.g., housing officers, to signpost or refer smokers to SSS.
■ Hospital staff to operate an opt-out referral system to SSS.

In deciding on the target behaviour, it was identified that a number of local interventions were already in place to support referrals to SSS from NHS and non-NHS professionals, but that few referrals came from the smokers themselves. Discussions with current smokers and ex-smokers suggested they would not consider booking a stop smoking service/advisor appointment as they did not know how to, did not want to make phone calls, or were not aware of the service or what it offered. Stop smoking advisors also revealed that a number of people attending an initial appointment do so with little motivation to stop smoking and often a lack of awareness as to why they have been referred by their GP or healthcare provider. As smokers who seek help themselves tend to be more successful at stopping than those recruited by others [9], we chose to design an intervention targeting two behaviours: (i) booking an initial SSS appointment and (ii) attending that initial SSS appointment.

#### 4.1.3. Step 3—Specify the Target Behaviour

Step 3 involves detailing the specifics of the behaviour, frequency, duration, and the context in which it needs to occur in order for the behaviour to be carried out (see Table 1). Being clear about this detail is important for fully drawing out the range of barriers to and facilitators of behaviour change in the next step.

#### 4.1.4. Step 4—Understanding the Target Behaviour and Understanding What Needs to Change

This step involves identifying what conscious and automatic cognitive processes in the individual or which factors in the environment need to change in order to achieve the desired change in behaviour. We explored the barriers to and facilitators of smokers’ capability, opportunity, and motivation (the components of the COM-B) to access (book) and attend a SSS. This included conducting a literature review of the existing evidence and collecting questionnaire-based data from current and ex-smokers. A more detailed exploration of the findings will shortly be published elsewhere.

##### Literature Review

A literature search of articles focusing on factors related to uptake of SSS revealed a number of perceived barriers identified by smokers. Practical barriers such as a lack of awareness about the service; the belief they did not have or know how to get access; that appointment choice would be limited; that they had no time to attend; and the financial cost of quitting were cited [25,26,27,28]. Psychological or belief-based barriers included fear about being judged, lectured, or nagged; fear of disappointing themselves or staff; seeing the need for stop smoking services as a weakness; and the perception that the service would not be effective [25,27,28,29,30,31].

A systematic literature review [29] found that identifying smokers from practice records, providing brief advice, screening tools, cold calling, and referral to SSS increased uptake and attendance. Self-referrals (the focus of this intervention) increased as a result of direct mail. Additionally, calls to quit lines from disadvantaged groups increased when social marketing techniques were employed.

##### Questionnaires with Smokers and ex-Smokers

An online questionnaire-based study (*n* = 40) was conducted to further explore the beliefs of smokers and ex-smokers who had and had not used SSS regarding the service and why they would or would not choose to access it. Outcomes were similar to the extant literature. The main barriers identified included a lack of knowledge about what the service is, the belief that booking would be difficult, and the belief that help from a service should not be needed or would not be helpful. Participants also believed they would be nagged and judged and felt that seeking help from a service is a sign of weakness. Additionally, they felt they would disappoint themselves and the service if they failed.

Data from the literature and questionnaires were subject to behavioural analysis, as outlined by the Behaviour Change Wheel framework. The findings aligned with ‘Capability’ (e.g., a lack of knowledge about the benefits of SSS), ‘Opportunity’ (e.g., beliefs that SSS are not easy to access), and ‘Motivation’ to act (e.g., beliefs that they did not need and would not benefit from SSS) within the COM-B model. These were further mapped onto the Theoretical Domains Framework (TDF) to provide an additional level of detail (See Table 2).

### 4.2. Stage 2—Identify Intervention Options

#### 4.2.1. Step 5—Identify Appropriate Intervention Functions

The BCW guides on which types of intervention functions are likely to initiate behaviour change in each COM-B component and associated TDF domain. Out of a possible nine intervention functions, the following six were identified as most useful for addressing the identified barriers within the context of an app (booking and attendance at SSS): (i) Education; (ii) Persuasion; (iii) Modeling; (iv) Incentivization; (v) Environmental Restructuring; and (vi) Enablement. Table 2 illustrates how the intervention functions relate to the corresponding COM-B and TDF components.

#### 4.2.2. Step 6—Identify Policy Categories

Three policy categories were identified as potential policy categories that could form the focus of an intervention: (i) ‘Communication/Marketing’; (ii) ‘Service provision’; and (iii) ‘Designing the physical/social environment’. Although the intervention is likely to include changes to these categories, this is more relevant to the promotion and delivery of the intervention than the intervention content *per se*. Therefore, this step was considered as a guide for post-intervention planning rather than intervention design.

### 4.3. Stage 3—Identify Content and Implementation Options

#### 4.3.1. Step 7—Identifying Behaviour Change Techniques (BCTs)

Behaviour change techniques (BCTs) linked to the relevant chosen interventions functions were identified using the Behaviour Change Technique Taxonomy (BCTTv1), which lists 93 BCTs with descriptions and examples of their application. Following training in the identification and coding of BCTs, and in accordance with BCW guidelines, the first three authors (E.F., K.B., and K.K.) independently generated a list of all possible BCTs aligned with the six selected intervention functions (see step 5). Then, through a process of discussion, the authors considered which BCTs could most feasibly be applied within the context of an app and would be most useful for addressing the identified barriers to the target behaviours of booking an appointment at, and attending SSS. In total the following 17 BCTs were identified as relevant: (1) Information about social/environmental consequences; (2) Salience of Consequences; (3) Social comparison; (4) Credible Source; (5) Comparative imagining of future outcomes; (6) Material incentive; (7) Vicarious Consequences: (8) Instruction on how to perform the behaviour; (9) Social Support (Unspecified); (10) Information about other’s approval; (11) Anticipated Regret; (12) Framing/Re-framing; (13) Identity associated with changed behaviour; (14) Focus on past success; (15) Verbal persuasion about capability; (16) Information about emotional consequences; and (17) Social reward.

**Figure 3:**
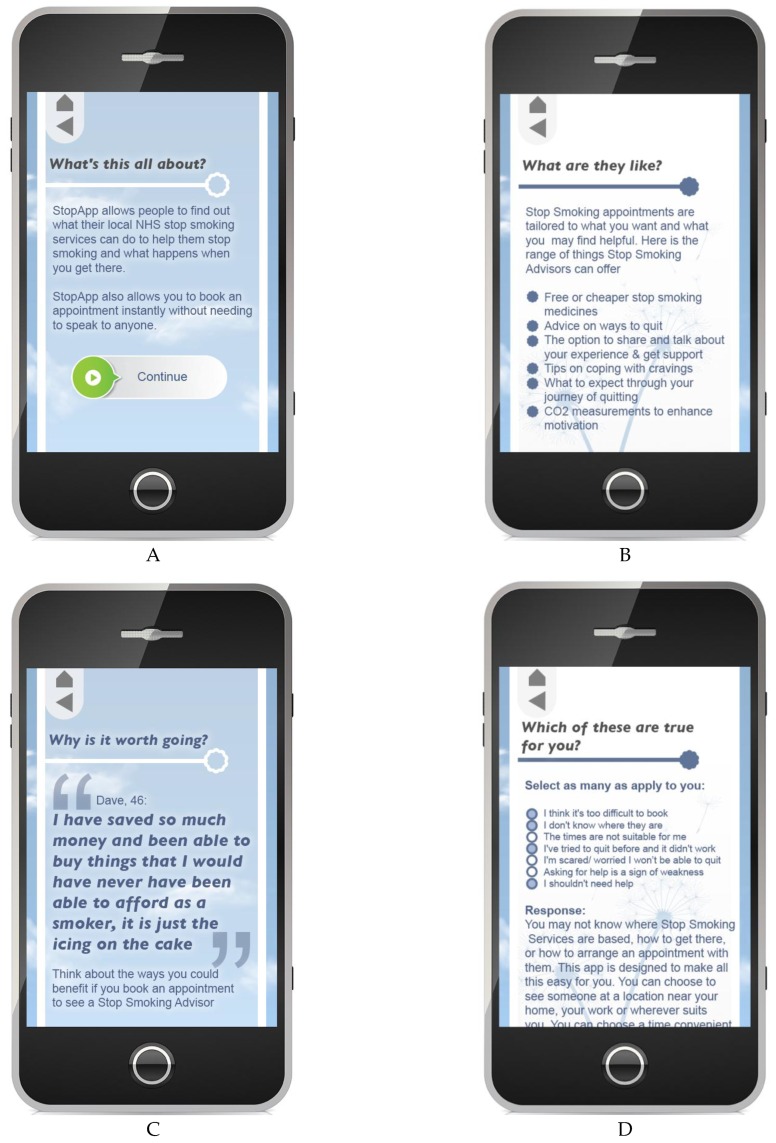
Screenshots from prototype app: (**A**) part of the introduction to the app; (**B**) information about the fact that SSS offer a range of approaches tailored to the user; (**C**) a genuine quote from an SSS user who successfully quit, with name and age; (**D**) screen that begins to provide tailored content for barriers relating to practical access to SSS and concerns about needing to access support to stop.

Once BCTs have been identified, content development requires some creativity. To help us develop a logical sequence and flow of content we considered the barriers to SSS access and uptake we had identified through literature review and our own data analysis. Barriers largely fell into one of three categories: (1) Concerns about ‘what services are like’ (e.g., judgmental, clinical, inflexible, not effective); (2) Practical concerns (e.g., not knowing how to book or access SSS, believing appointments will be inconvenient); (3) Feelings related to you needing to stop without help and fear of failure if attempts are made to stop. We therefore developed content around these themes. Users are initially presented with content that deals with ‘what services are like’; they can then select further content aligned with either practical concerns and/or beliefs associated with it being wrong to need help to stop, as relevant to them (see Figure 3D below). Content is also tailored by age and gender. We have access to and permission to use real SSS users’ comments about their experiences and will match content as closely as possible with the age and gender of the user (e.g., see Figure 3C below). As content ideas were developed we continually referred to our selected BCTs to ensure the content applied to them. We will continue to do this throughout technical development and user testing going forward to ensure the fidelity to the BCT content. See Table 3 below for example BCTs and their operationalization within StopApp. See Figure 3 for example prototype screen shots of the content under development within the app.

#### 4.3.2. Step 8—Determining the Mode of Delivery

A digital intervention was chosen as the mode of delivery, and agreed with the expert panel at the outset of the project. We decided to develop a web app rather than a standard app so that it could be viewed from both mobile phones and computers. In order to fully assess the feasibility of this and views of the SSS, we conducted telephone interviews with health professionals and Stop Smoking Advisors working in both NHS and non-NHS settings, and also non-NHS community services, to explore their views about such an intervention and how it could be promoted and delivered.

##### Telephone Interviews with Stop Smoking Advisors

Stop Smoking Advisors (SSAs) in both NHS and non-NHS sites (e.g., independent pharmacies) were interviewed (*n* = 6) to assess the acceptability of the proposed intervention. The SSAs reported that a digital intervention would offer something extra to the current service and the concept of instant online booking also met with approval. In particular, they thought that the opportunity to book quickly, easily, and privately would act as a facilitator to access. They argued that this along with the behaviour change element of the intervention could facilitate people to capitalize on their own motivation, which is particularly important for people self-referring to the service, the main route to pharmacy-based SSS. They particularly liked the idea that the app will address *what to expect* at the service, *what will happen*, and *what can be offered*.

##### Telephone Interviews with Non-NHS Community Services

Representatives from the local children’s center, the library service, leisure service, Wellbeing Hubs (MIND), and Citizen’s Advice Bureau were interviewed regarding their views about the proposed intervention and mode of delivery via an app. Services reported they would be very interested in promoting the app and suggested a number of ways they could support service users to use it. For example, the library service was happy to have a link on their desktop computers and provide staff support for use. The children’s centers described how they had access to iPads that could be used to help mothers use the StopApp. The leisure centers suggested use of their existing marketing and promotion schemes to raise awareness about the app, particularly to more deprived communities. Findings from the interviews will be used to consider the best path for implementation of the app within service provision. This will be further explored in a future evaluation of the app in different non-NHS settings.

The draft content, layout, appearance, and functionality of the StopApp have been reviewed and critiqued by members of our Patient and Public Involvement (PPI) group, which is made up of current and ex-smokers. This is to ensure that it is appropriately worded and pitched, that it delivers the BCTs appropriately and accurately, and that the app is engaging and easy to use. A near-final version of the app will undergo full end-user testing with our PPI group and expert panel to refine and test the app’s features and content.

### 4.4. The StopApp Intervention

The StopApp intervention is designed to increase motivation to attend, booking, and attendance at SSS. It includes a brief evidence-based, tailored behaviour change intervention consisting of 17 BCTs designed to encourage booking an appointment at SSS and attendance. This is supplemented by an instant online booking system to provide an opportunity for performing the desired behaviour when motivation is increased. Users can book at any stage in their use of the app by clicking on a link running along the bottom of each page. The StopApp content is designed to engage users with the use of infographics and user stories rather than heavy text. Messages are tailored to the user’s age, gender, and beliefs about the barriers to attending SSS. StopApp can be delivered to end-users in web app format via their personal mobile phone, desktop computer, or computers/tablets used in various NHS and community settings.

Instant booking has been developed for pharmacy-based SSS appointments via links to the existing live booking system used in Warwickshire so that information is securely and instantly sent to the service and not stored on the app. Only names and telephone numbers are input into the StopApp, enabling SSS staff to cancel or rearrange appointments if required. To further support attendance, users of StopApp are sent SMS confirmation and reminders to attend their appointment, along with advice about how to get there and parking should they request it. For examples of how the BCTs are represented in the app, please see Table 3 and Figure 3.

## 5. Discussion

StopApp is a novel theory-driven and evidence-based intervention that aims to increase referrals to and attendance at SSS. Secondary outcomes may include increased four-week quit rates. It includes a brief evidence-based behaviour change intervention designed to increase motivation to book and attend SSS, including the facility to instantly book an appointment, providing opportunity when motivation is increased and reminders to attend. Recent research suggests that smokers who reported higher motivation to quit on attending SSS had the highest quit rates; this was not influenced by gender or level of deprivation, which suggests the importance of motivation as a driver of behaviour change [32]. Reducing wasted appointments and administrative time relating to arranging and booking appointments could lead to significant cost savings. More smokers setting a quit date and ultimately stopping smoking will undoubtedly carry significant cost savings in terms of healthcare utilization.

The StopApp is based on a theoretical framework of behaviour change and draws on evidence regarding the barriers to uptake at SSS. This in-depth approach to intervention development increases the likelihood that it will effectively achieve increased uptake over and above that of other existing methods such as promotional campaigns, invitation letters, and referrals from health professionals and staff from other community services. We intend to test whether this is the case by comparing levels of uptake from the StopApp with a standard letter of invitation to attend SSS and a leaflet about the service.

There is a need to ensure we reach disadvantaged groups in the choice of intervention. Many people rarely use NHS services and therefore may miss the opportunity to be referred or to discover what SSS can offer. StopApp can be widely implemented and is scalable nationally, impacting on harder-to-reach groups if advertised in pubs, community centers, and workplaces, for example. Although suggested in the literature, the relative cost savings of using web apps is not clear. Costs will include development and app design, hosting, and sending text messages and promotional materials to advertise the app. Existing methods to increase uptake at SSS include referrals from professionals on behalf of the smoker, which carry a cost in terms of the time to make the referral, postage costs if sending the referral is not electronic, and the administrative cost associated with a stop smoking advisor telephoning the smoker to arrange an appointment. StopApp incorporates an e-booking system, therefore removing these costs. By encouraging more motivated smokers to book and sending text message reminders to attend, the prediction is that there will be fewer wasted appointments associated with bookings from the StopApp, and those who do attend will be more motivated to set a quit date and to stop smoking. App-based interventions are easy to promote and access via non-NHS settings, via Public Health services already involved in improving health and wellbeing. The app could be accessed independently via promotional posters with QR codes to enable download, via websites, and also via direct access on electronic devices within services (e.g., computers in libraries). Exploring the feasibility of the setting for delivery, and who delivers it, will be a key component of a future full trial.

### A Web App Intervention

A number of studies have evaluated the use of apps and websites as a smoking cessation aid (e.g., QuitText [33]; StopAdvisor [34]; iQuit [35]). However, to the authors’ knowledge none have explored them as a method of increasing uptake at SSS. An ongoing NIHR-funded trial, ‘Start2Quit’ [15], is currently evaluating the impact of “personal tailored risk information and taster sessions” to increase uptake at SSS. This utilizes a proactive recruitment approach; however, it is letter-based and does not include a choice of SSS time and location and an e-referral facility. App-based interventions are also more likely to be shared between peers via social media and e-mail [36].

## 6. An Instant Booking System

Existing e-referral systems for booking healthcare appointments online include the historic ‘Choose & Book’ system, and the recently launched ‘Patient Booking Website’ for NHS e-referral services. It is unclear whether SSS bookings will be possible; however, the system still requires a GP referral and may not reach more disadvantaged groups who access NHS services less frequently. The odds of quit attempts are increased with increased levels of motivation at the baseline [37]. Motivation to act changes over time, and even if an intervention increases motivation to book an appointment at SSS, a time lapse between agreement to be referred to SSS and making or receiving a call from an advisor could be enough to lose motivation and intention to attend [26]. Therefore, the ability to book an appointment at a point when motivation is high could lead to improved outcomes. Removing administrative barriers to referring people to stop smoking services could also increase referral rates. The National Centre for Smoking Cessation Training (NCSCT) Stop Smoking Referral System provides a system for staff in hospitals to refer patients to SSS by accessing relevant records automatically and sending referrals electronically. The system was designed to be used in conjunction with staff offering ‘Very Brief Advice’ (VBA) as part of Making Every Contact Count (MECC) [38]. MECC is a national program designed to make use of the millions of everyday contacts staff (both NHS and non-NHS) have with patients and the public as an opportunity to raise the issue of healthy lifestyle change and signpost available support. A three-month trial in Portsmouth resulted in a 600% increase in referrals to SSS [39]. However, the system is only available within hospital settings and requires health professional input to know who to target to start the conversation. In a meta-analysis, SMS reminders were found to substantially increase attendance at healthcare appointments [40]. The cost of non-attendance at NHS outpatient appointments is estimated at £790 million p.a. [41]. Adjusted for inflation, this 2005 figure is now over £1 billion. Therefore, the inclusion of text reminders to attend in StopApp is likely to lead to even greater attendance and cost savings by reducing missed appointments (DNAs). StopApp has the potential to increase attendance at SSS by increasing motivation to book, removing the barriers to attendance, enhancing the ease of booking, and reminding people to attend.

## 7. Conclusions

The potential outlined above for StopApp must be assessed. In the next phase of the research, we will conduct a full trial and health economic evaluation to assess the effectiveness of the StopApp at increasing booking and attendance at services, reducing wasted appointments, and improving outcomes (four-week quit rates) by virtue of a more motivated self-selected sample of smokers who have proactively chosen to be there. If effective, the StopApp could be adapted for use by local authorities nationally to enhance uptake and outcomes in SSS, in order to help reduce the prevalence of smoking in the United Kingdom. StopApp has the potential to enhance MECC activity both within and outside of the NHS, reducing the burden on service staff and providing an intervention that could reach more disadvantaged and less accessible groups, supporting them to access help to stop smoking.

## Figures and Tables

**Table 1:** Specifying the target behaviour and content.

Target Behaviour	Smokers to Book and Attend an Initial Appointment at a Stop Smoking Service in Warwickshire
Who needs to perform the behaviour?	Anyone who smokes
What do they need to do differently to achieve the desired change?	They need to know that the stop smoking service exists
	They need to know how to book an appointment
	They need the opportunity to book an appointment
	They need to understand what the stop smoking services offers
	They need to know when and how to attend an appointment
When do they need to do it?	As soon as motivation/intention to stop smoking is increased
Where do they need to do it?	Anywhere they have access to the intervention via mobile phone, tablet or computer.
How often do they need to do it?	Once to book and once to attend initial appointment. It is then the role of the SSS to encourage continued attendance
With whom do they need to do it?	On their own or with support from a health/community professional

**Table 2:** Examples of how the findings from the behavioural analysis mapped onto the Behaviour Change Wheel.

COM-B		Relevant TDF	Description of What Needs Addressing in the Intervention Based on Data Collected/Literature	Intervention Functions	Policy Categories	Behaviour Change Techniques (BCTs) Identified
Capability	Psychological Capability	Knowledge	1. A lack of knowledge about the benefits of the service—knowledge about what it does, how it has helped others, what it offers beyond what people may already know, that you are offered more than one appointment.	Education Persuasion Modeling Incentivization (NRT)	Communication/Marketing Service provision Designing the physical/social environment	5.2 Salience of Consequences 5.3 Information about social/environmental consequences 6.2 Social comparison 9.3 Comparative imagining of future outcomes 10.1 Material incentive 16.3 Vicarious Consequences
			2. A lack of knowledge about the ethos (approach), non-judgmental, not just about health risks, supports realistic expectations, time is offered for support (not just quick prescription), how it engages people.	Education Persuasion Modeling		5.3 Information about social/environmental consequences 6.2 Social comparison
			3. A lack of knowledge about access—knowing the service exists, where it is, when you can go, how to get an appointment.	Education Enablement Environmental restructuring		4.1 Instruction on how to perform the behaviour
Opportunity	Physical Opportunity	Environmental context and resources	Perception that time is a barrier- finding the time and the availability of appointments at correct time. Perception that SSS are not easy to access Mode of delivery of invitation—want to be invited face to face (app will be, but not part of content *per se*) Ease and privacy of booking via app	Enablement Education Environmental restructuring		4.1 Instruction on how to perform the behaviour 7.1 Prompts/cues
	Social Opportunity	Social influences	Perception that no one has used SSS successfully Stigma and blame Others around you Encouragement from health professionals	Education Persuasion Modeling Environmental restructuring		5.3 Information about social/environmental consequences 5.6 Information about emotional consequences 6.2 Social Comparison 6.3 Information about others’ approval 11.2 Reduce negative emotions
Motivation	Reflective Motivation	Professional/social role and identity	Don’t like the idea of needing or seeking help/belief that smokers should quit on their own Using SSS is a sign of weakness I can do it if I want/need to Disappointment with self if failed	Education Persuasion		5.5 Anticipated Regret 5.6 Information about emotional consequences 11.2 Reduce Negative Emotions (see example below) 13.2 Framing/Re-framing 13.5 Identity associated with changed behaviour 15.3 Focus on past success
		Beliefs about capabilities	Don’t need support/help/SSS to successfully give up smoking—Already aware of the risks, have quit before, Would access services if was unsure could do it alone	Education Persuasion		5.3 Information about social/environmental consequences 5.6 Information about emotional consequences
		Optimism	1. I don’t need help—Unrealistic Optimism	Education Persuasion Modeling		6.2 Social Comparison 15.1 Verbal persuasion about capability
		Beliefs about consequences	Knowing what to expect, what support, after ‘treatment’, how they would help, more than just NRT, time of appointments, confidentiality Expectation they will be nagged, fear of being judged. SSS don’t offer more than they already know Lack of knowledge of successful outcomes Non-committal—not committed to stopping smoking just the appointment Not knowing if you could go back if needed Beliefs that SSS don’t work—ineffective, it will not help me stop smoking	Education Persuasion Modeling		5.3 Information about social/environmental consequences 5.6 Information about emotional consequences 6.2 Social Comparison 13.2 Framing/Re-framing
		Intentions	N/A			
	Automatic Motivation	Reinforcement	Lack of knowledge about free prescriptions —would go to SSS if they offered incentives such as free prescriptions, *etc*.	Education Incentivization		10.1 Material incentive
		Emotion	Fear of failing—the fear of failing stops me accessing SSS, it’s too difficult, disappointed in self if fail. Sign of weakness	Education Modelling Persuasion Incentivization		5.6 Information about emotional consequences 10.5 Social reward 11.2 Reduce negative emotions 5.2 Salience of consequences 13.2 Framing/reframing

**Table 3:** Examples of some of the BCTs included in the StopApp and related detail about their content/representation, including reference to figures showing example prototype screenshots. ^1^

BCT #	BCT Label	BCT Definition	Examples of How this Is Represented in the App
**2**	**4.1 Instruction on how to perform the behaviour**	*Advise or agree on how to perform the behaviour (includes ‘Skills training’)*	Instructions in the app about how to find a service near home or work, how to then choose a date/time, and how to book. Information on how to attend (location, how to get there, *etc*.) (presented in Figure 3D)
**9**	**6.2 Social comparison**	*Draw attention to others’ performance to allow comparison with the person’s own performance*	Stories from peers about success at SSS, not being judged, given right support, offered more than could do alone.
**11**	**7.1 Prompts/cues **	*Introduce or define environmental or social stimulus with the purpose of prompting or cueing the behaviour*	Information about and provision of text reminders to attend appt. Option of support to make a plan to help people to get there
**13**	**9.3 Comparative imagining of future outcomes**	*Prompt or advise the imagining and comparing of future outcomes of changed versus unchanged behaviour*	Positive stories from peers about life after smoking with prompt to consider own future outcomes (see Figure 3C)
**14**	**10.1 Material incentive**	*Inform that money, vouchers, or other valued objects will be delivered if and only if there has been effort and/or progress in performing the behaviour*	Give the message that they can get free NRT from SSS (see Figure 3A).
**15**	**13.2 Framing/Re-framing**	*Suggest the deliberate adoption of a new perspective on behaviour (e.g., its purpose) in order to change cognitions or emotions about performing the behaviour*	Stopping smoking is hard; even if do not want emotional support, SSS may provide NRT or practical ideas. Address belief that it is difficult/not practical to go—actually easy to book and access (partly addressed in Figure 3A,D)
**18**	**15.3 Focus on past success**	*Advise thinking about or listing previous successes in performing the behaviour (or parts of it)*	The message that stopping smoking for any period is a success. Even applicable to periods of not smoking that are unintentional (e.g., long-haul flight). Each attempt is one step closer. Can learn a lot from previous attempts to quit that will help with stopping for good

^1^ The final BCT content of the StopApp will only be coded and agreed once full app development and user testing has taken place with input from smokers and ex-smokers. # means “number”, refers to the numbered list in the paragraph listing the BCTs above.
